# Supplementary material for: Bi-Allelic Mutations in Zebrafish pank2 Gene Lead to Testicular Atrophy and Perturbed Behavior without Signs of Neurodegeneration
Source: Int J Mol Sci. 2022 Oct 26;23(21):12914. doi: 10.3390/ijms232112914 (PMC9657214; doi:10.3390/ijms232112914)
Supplement: Supplementary file 1 [file ijms-23-12914-s001.zip › ijms-1879217-supplementary.pdf]

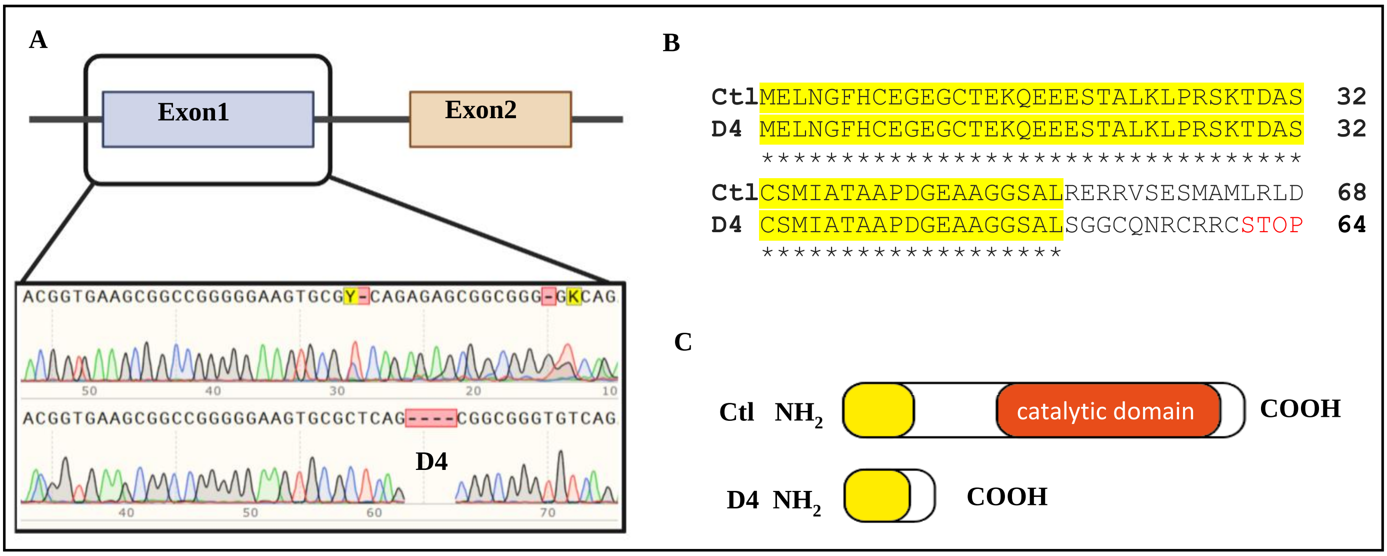

Supplementary Figure S1. Description of the CRISPR/Cas9-induced mutation in *pank2* gene (**A**) Chromatograms (Sanger sequencing) of D4 and wild-type *pank2* exon 1. (**B**) Alignment of the wild-type amino acid sequence with the predicted D4 mutant sequence, obtained by the Expasy translate tool (https://web.expasy.org/translate/). (**C**) Graphic representation of the predicted mutant protein; the yellow area indicates conserved amino acids; the white one different amino acids.

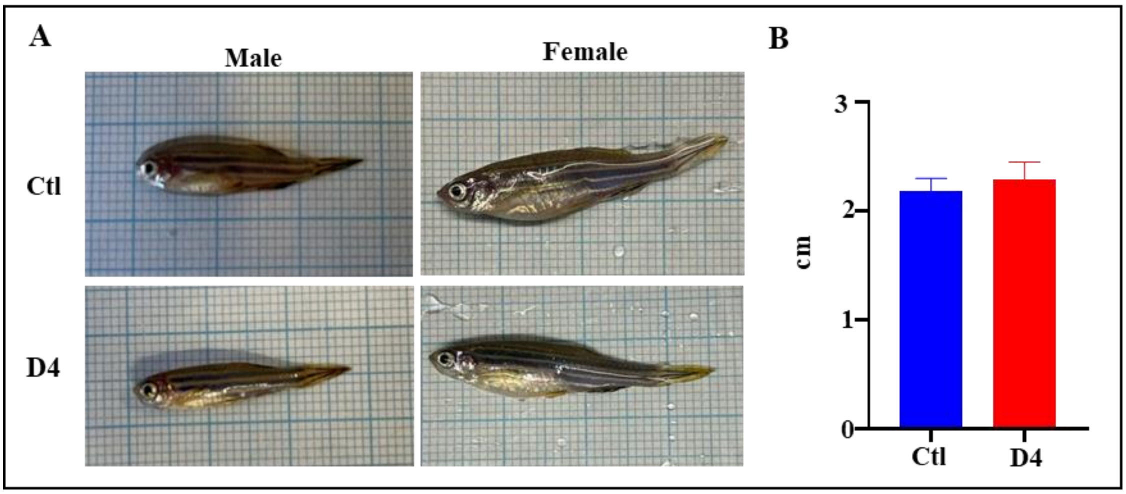

Supplementary Figure S2. Morphological characterization of 1-year-old D4 mutant fish. (**A**) Lateral view of representative 1-year-old adult mutant fish on graph paper. Left panels = females. Right panels = males. (**B**) Measurement of total body length of adult fish; N = 8/group.

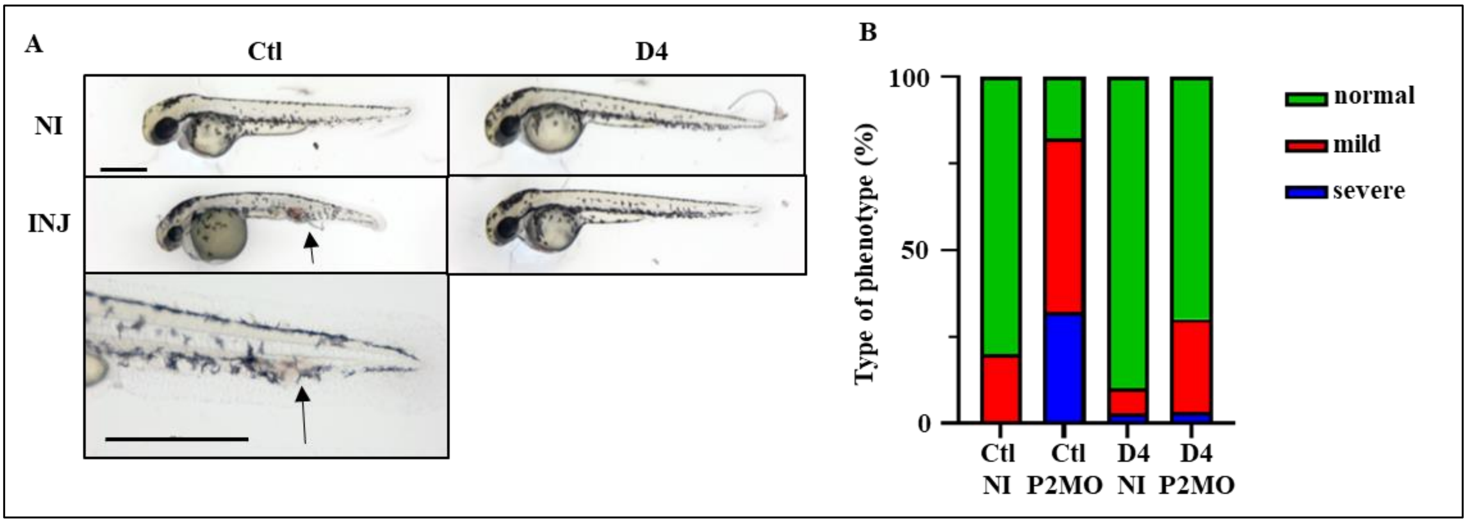

Supplementary Figure S3. Mutant line validation by injection of a *pank2*-specific morpholino (P2MO). (**A**) Lateral view of 48 hpf embryos; arrows indicate blood accumulation. (N = 1, D4 NI = 50 embryos, D4 P2MO = 63 embryos, Ctl NI = 57 embryos, Ctl P2MO = 59 embryos); Size bar = 500 µm. (**B**) Graph with the phenotype distribution in not injected and injected embryos. NI, not injected; P2MO, pank2-specific morpholino-injected embryos.

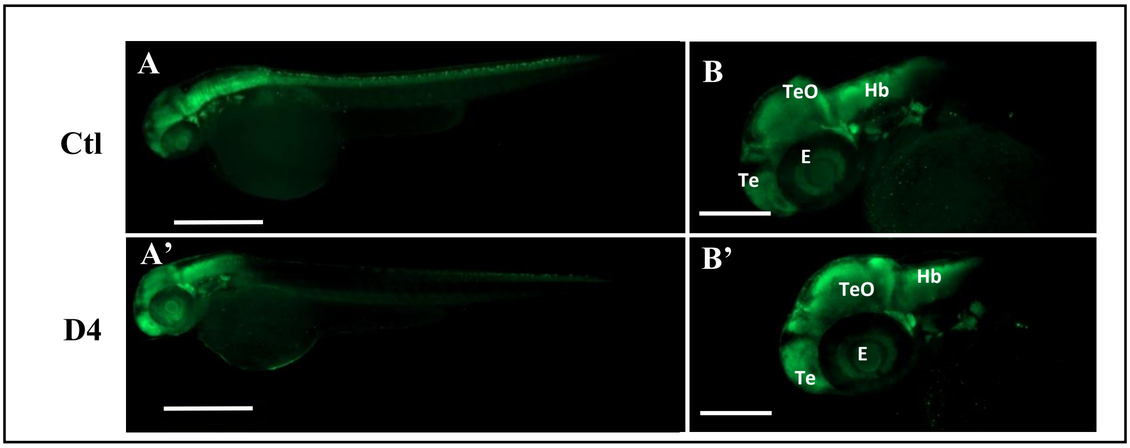

Supplementary Figure S4. Immunofluorescence staining for the pan-neuronal marker Elavl3+4. (**A, A'**) Lateral view of stained embryos at 48 hpf. N = 2; D4 (37/40) and control embryos (37/38). Size bar = 500µm. (**B, B'**) Magnification of the lateral views of the head of 48 hpf embryos. E, eye; Hb, hindbrain; Te, telencephalon, TeO, tectum opticum.

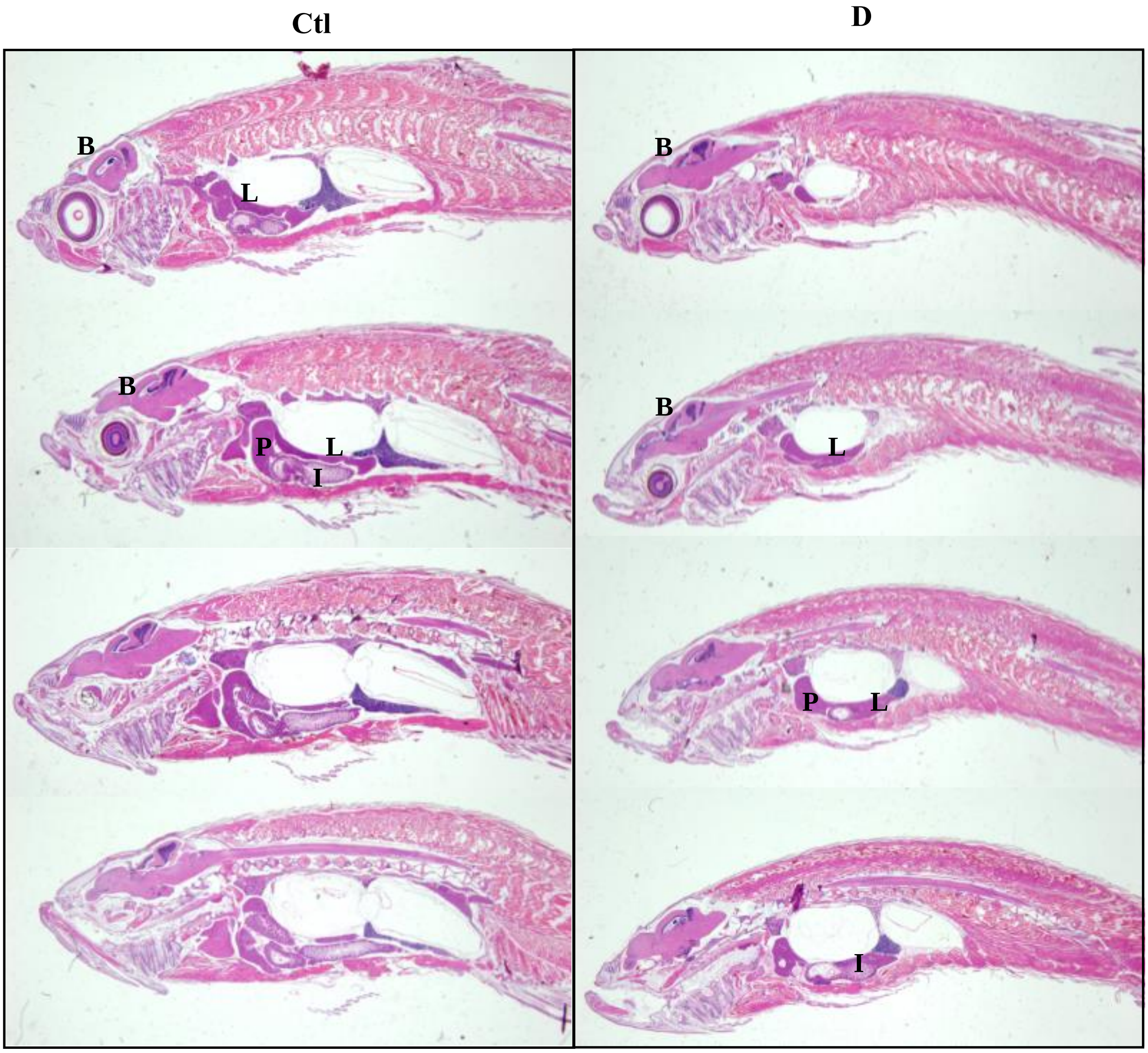

Supplementary Figure S5. Histological sections stained with hematoxylin-eosin of 1-year-old fish. B, brain; I, intestine; L, liver; P, pancreas. N = 7 for each group

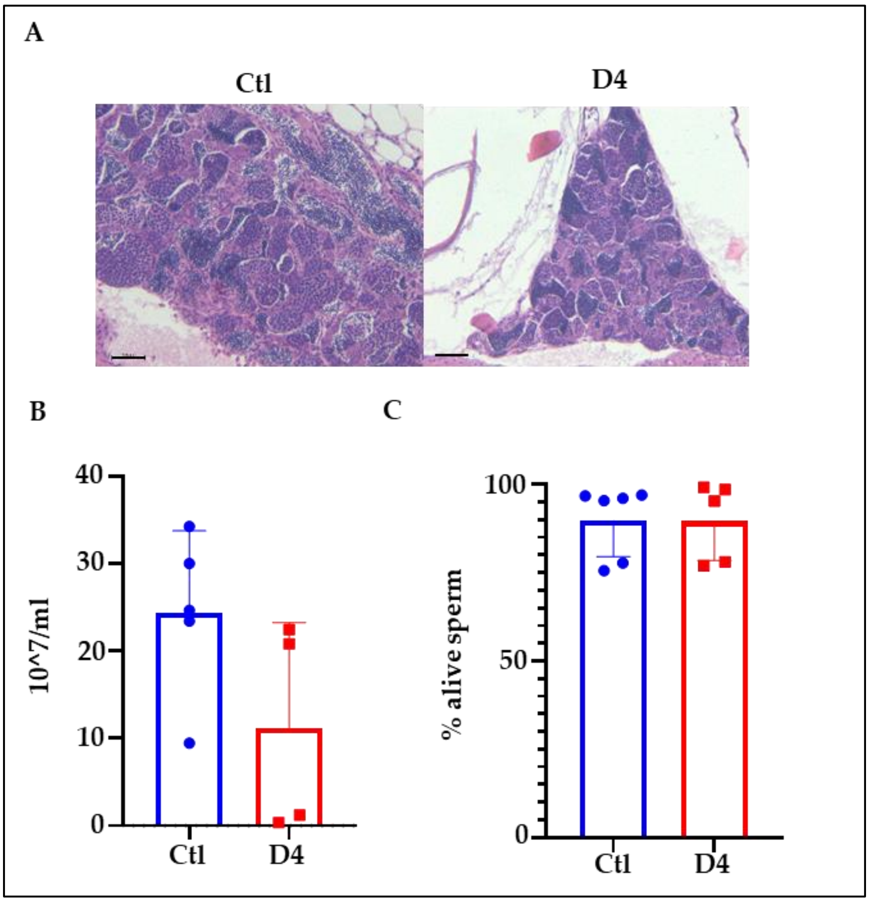

Supplementary Figure S6. Number and vitality of spermatozoa in 1-year-old fish. (A) Representative histological sagittal sections of 1-year-old fish, stained with hematoxylin-eosin. N = 5 for each group. Magnification 110X. Size bar = 50 µm. (**B**) Concentration of ejaculated sperm from 1-year-old fish. Ctl = 5, D4 = 4; Ctl mean = 24.35*10^7/ml, D4 mean = 11.20*10^7/ml. (**C**) Viability of ejaculated sperm from 1-year-old fish. Ctl = 5, D4 = 5; Ctl mean = 89.90%; D4 mean = 89.66%.

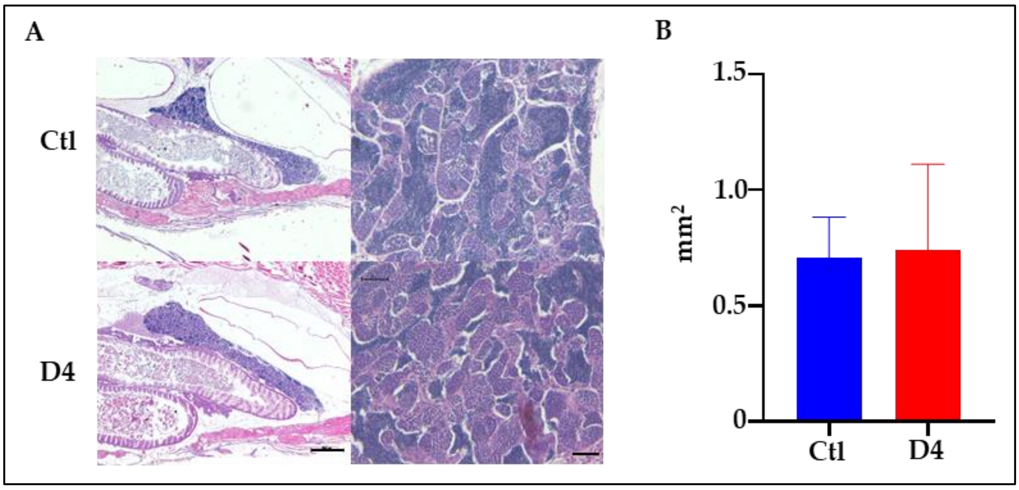

Supplementary Figure S7. Investigation of the testis in 3-month-old fish. (**A**) Representative histological sagittal sections of 3-month-old fish, stained with hematoxylin-eosin. N = 3 for each group. Magnification 40X and 110X. Size bar = 500 µm and 50 µm (**B**) Quantification of testis area performed on histological sections; Ctl mean = 0.70 mm^2; D4 mean = 0.74 mm^2.

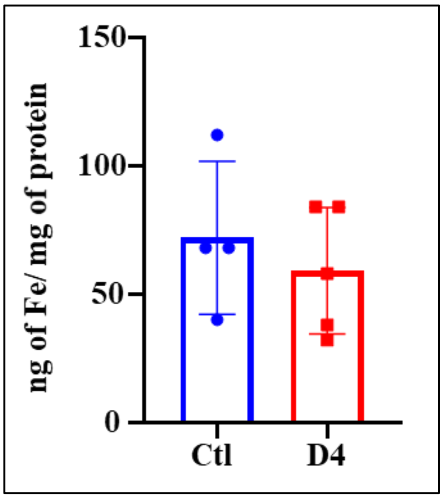

Supplementary Figure S8. Iron quantification in the brain. Iron quantification by ICP-MS in brains from 1-year-old control (Ctl).and mutant (D4) fish. N = 4 for controls and N = 5 for D4.

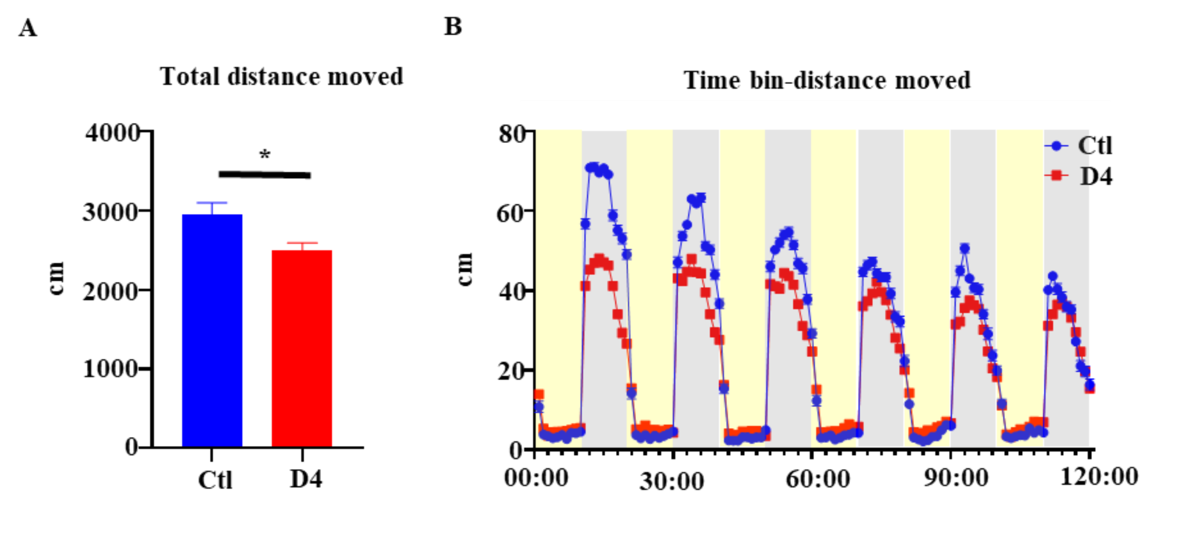

Supplementary Figure S9. Behavioral study in control and mutant embryos at 5 dpf. (**A**) The graph shows the mean plus standard deviation of the total distance swam by control (Ctl) and mutant (D4) fish. (**B**) Time bin analysis of the movement. Yellow indicates light on, gray indicates light off. Ctl = 67, D4 = 125, two biological replicates. * $P < 0.05$. Student's t-test.

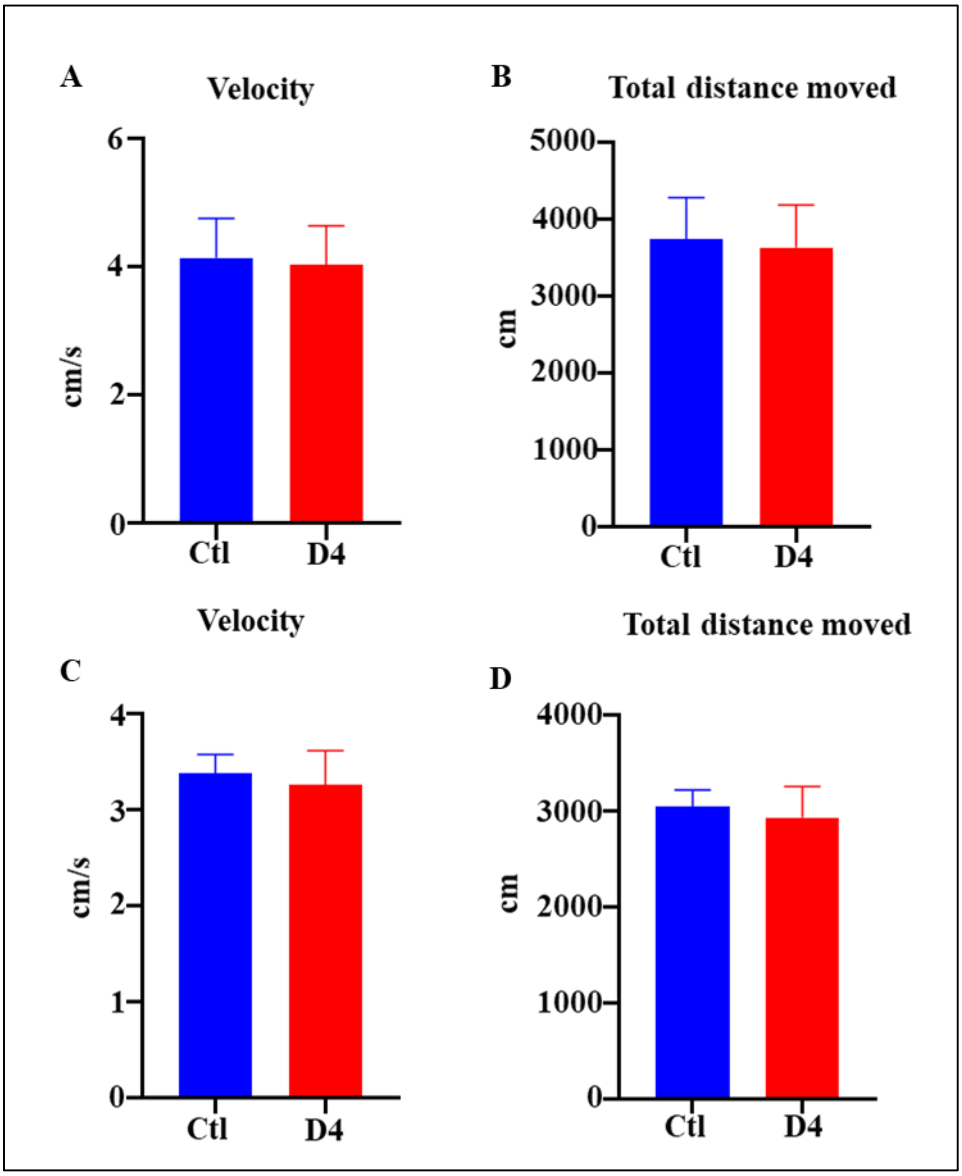

Supplementary Figure S10. Behavioural analysis in adult fish. (**A**) Total distance swam and (**B**) mean velocity of 1-year-old fish; N = 11 for Ctl and 12 for D4. (**C**) Total distance swam and (**D**) mean velocity of 3-month-old fish. N = 3.

qPCR primers		
Gene		Sequence
coasy	Forward:	5’- CATTGGCTCTTCAGTCTCCTC-3’
coasy	Reverse:	5’- AGGTTTGGGTCGCAACTATC-3’
pank2	Forward:	5’- TCCATGAAGTTGCTCGCGTA-3’
pank2	Reverse:	5’- CAGCTCAGGATGGTTTGGT-3’
pank1a	Forward:	5'-TACCTCACGTCCAACACAGC-3'
pank1a	Reverse:	5'-AGTCAATGTACAGCAGGCCC-3'
pank1b	Forward:	5'-CGGAGGTGGTCTGGGTACTA-3'
pank1b	Reverse:	5'-CGGCCCATTTGGATAAAGCG-3'

Genotyping		
Gene		Sequence
pank2	Forward:	5-AGCTGCCTCGGTCAAAGAC-3'
pank2	Reverse:	5'-AGTGAGTCGAGCCTCAGCAT-3'

sgRNA		
Gene		Sequence
pank2		GGGGAAGTGCGCTCAGAGAG

Supplementary Table S1. Sequences of primers and sgRNA.
